# Supplementary material for: Lipidomics of human adipose tissue reveals diversity between body areas
Source: PLoS One. 2020 Jun 16;15(6):e0228521. doi: 10.1371/journal.pone.0228521 (PMC7297320; doi:10.1371/journal.pone.0228521)
Supplement: S1 Method — (DOCX) [file pone.0228521.s001.docx]

### Supplementary Method

**Detailed description of LC-MS method:**

The samples were analyzed in random order using an in-house routine lipidomics platform utilizing an ultra-high-performance liquid chromatography quadrupole time-of-flight mass spectrometry method (UHPLC-Q-TOF-MS) in positive and negative electrospray ionization mode.

The samples were separated on a ACQUITY UPLC BEH C18 column (2.1 mm x 100 mm, 1.7 *µ*m) by Waters (Milford, CT, USA) fitted with a VanGuard (Waters pre-column) on a 1290 infinity UHPLC system (Agilent, Santa Clara, CA). This was equipped with a multisampler (maintained at 10⁰C), a quaternary solvent manager and a column thermostat (maintained at 50⁰C). 10% dichloromethane (DCM) in methanol (MeOH) and acetonitrile:methanol:isopropanol:water (ACN:MeOH:IPA:H_2_O) (1:1:1:1, v/v/v/v)+0.1% formic acid (HCOOH) was used as wash solutions after each injection for 7.5 s each. The UHPLC system was connected to a 6550 iFunnel quadrupole time-of-flight mass spectrometer (Q-TOF-MS) with a dual jet stream electrospray (dual ESI) ion source from Agilent Technologies (Agilent).

To achieve chromatographic separation of the lipids, 1 µl of each sample was injected on the column. Eluent A was consisted of (H_2_O + 1% 1M ammonium acetate (NH4Ac) + 0.1% HCOOH) and eluent B of (1:1 ACN:IPA + 1% 1M NH4Ac+ 0.1% HCOOH). The flow rate was kept constant at 0.4 ml/min. and the following gradient elution was used: from 0 to 2 min 35-80% B, from 2 to 7 min 80-100% B and from 7 to 14 min 100% B. Each run was followed by a 7 min re-equilibration period under initial conditions (35% B). Nitrogen generated by a nitrogen generator (PEAK Scientific, Renfrewshire, Scorland, UK) was used as the nebulizing gas at of 21 psi, as the drying gas with a flow rate set to 14 L/min at 193⁰C and as the sheath gas with a flow rate set to 11 L min^-1^ at 379⁰C. For collision gas, pure nitrogen (6.0) from Praxair (Fredericia, Danmark) was used. The optimized MS conditions were as follows: capillary voltage and the nozzle voltage were maintained at 3643 and 1500 V, respectively. Reference mass solution (including ions at m/z 121.0509 and 922.0098) was prepared according to instructions by Agilent and it was introduced to the mass spectrometer using a separate Agilent series 1290 isocratic pump at a constant flow rate of 4 ml min-1 (split to 1:100 before the nebuliser). The acquisition mass range was *m/z* 100-1700 and the instrument was run using the extended dynamic range with a resolution of 30000 FWHM measured at *m/z* 1521.9715 during calibration of the instrument. All data were acquired using MassHunter B.06.01(Agilent).
