# Supplementary material for: Lipidomics of human adipose tissue reveals diversity between body areas
Source: PLoS One. 2020 Jun 16;15(6):e0228521. doi: 10.1371/journal.pone.0228521 (PMC7297320; doi:10.1371/journal.pone.0228521)
Supplement: S1 Table — (DOCX) [file pone.0228521.s002.docx]

***Table 1****. Identified lipids from positive ionization mode.*

| **Lipid Name** | **Ionization Mode** | ***Mass-to-charge ratio (m/z)*** | **Retention time (RT)** |
| --- | --- | --- | --- |
| DG(34:0) | Positive | 614.57 | 8.32 |
| DG(36:0) | Positive | 642.60 | 8.64 |
| DG(36:1) | Positive | 640.59 | 6.77 |
| DG(40:6) | Positive | 686.57 | 4.61 |
| HexCer(d40:1) | Positive | 784.66 | 8.27 |
| LPC(15:0) | Positive | 482.32 | 3.81 |
| LPC(16:0) | Positive | 496.34 | 4.04 |
| LPC(18:0) | Positive | 524.37 | 4.51 |
| LPC(18:1) | Positive | 522.35 | 4.03 |
| PC(30:0) | Positive | 706.54 | 6.83 |
| PC(32:0) | Positive | 734.57 | 7.29 |
| PC(33:0) | Positive | 748.59 | 7.52 |
| PC(33:0) | Positive | 748.58 | 8.21 |
| PC(34:1) | Positive | 760.58 | 7.29 |
| PC(34:2) | Positive | 758.57 | 6.90 |
| PC(36:0) | Positive | 790.63 | 8.09 |
| PC(36:2) | Positive | 786.60 | 7.34 |
| PC(36:3) | Positive | 784.58 | 6.92 |
| PC(36:4) | Positive | 782.56 | 6.77 |
| PC(36:5) | Positive | 780.55 | 6.41 |
| PC(38:5) | Positive | 808.58 | 6.76 |
| PC(P-34:1)/PC(O-34:2) | Positive | 744.59 | 7.48 |
| PC(P-34:2)/PC(O-34:3) | Positive | 742.58 | 7.10 |
| PC(P-36:3)/PC(O-36:4) | Positive | 768.58 | 7.08 |
| PE(36:3) | Positive | 742.53 | 7.84 |
| PE(O-38:6)/PE(P-38:5) | Positive | 750.54 | 7.12 |
| PE(O-40:7)/PE(P-40:6) | Positive | 776.55 | 7.47 |
| SM(d34:1) | Positive | 703.57 | 6.88 |
| SM(d36:1) | Positive | 731.60 | 7.39 |
| SM(d37:1) | Positive | 745.62 | 7.60 |
| SM(d38:1) | Positive | 759.63 | 7.81 |
| SM(d42:2) | Positive | 813.68 | 8.13 |
| TG(40:0) | Positive | 712.64 | 8.85 |
| TG(40:1) | Positive | 710.63 | 8.55 |
| TG(42:0) | Positive | 740.67 | 9.14 |
| TG(42:1) | Positive | 738.66 | 8.81 |
| TG(42:2) | Positive | 736.64 | 8.53 |
| TG(44:0) | Positive | 768.70 | 9.44 |
| TG(44:1) | Positive | 766.69 | 9.10 |
| TG(44:2) | Positive | 764.67 | 8.81 |
| TG(45:0) | Positive | 782.72 | 9.58 |
| TG(45:1) | Positive | 780.70 | 9.22 |
| TG(46:0) | Positive | 796.74 | 9.77 |
| TG(46:1) | Positive | 794.72 | 9.39 |
| TG(46:2) | Positive | 792.70 | 9.08 |
| TG(46:3) | Positive | 790.69 | 8.79 |
| TG(47:1) | Positive | 808.74 | 9.51 |
| TG(47:2) | Positive | 806.72 | 9.20 |
| TG(48:1) | Positive | 822.75 | 9.72 |
| TG(48:2) | Positive | 820.74 | 9.36 |
| TG(48:3) | Positive | 818.72 | 9.05 |
| TG(49:0) | Positive | 838.78 | 10.27 |
| TG(49:1) | Positive | 836.77 | 9.84 |
| TG(49:2) | Positive | 834.75 | 9.49 |
| TG(49:3) | Positive | 832.74 | 9.18 |
| TG(49:3) | Positive | 832.73 | 10.14 |
| TG(50:0) | Positive | 852.79 | 10.55 |
| TG(50:1) | Positive | 850.78 | 10.07 |
| TG(50:2) | Positive | 848.77 | 9.67 |
| TG(50:3) | Positive | 846.75 | 9.33 |
| TG(50:4) | Positive | 844.74 | 9.04 |
| TG(50:5) | Positive | 842.72 | 8.81 |
| TG(50:5) | Positive | 842.72 | 9.83 |
| TG(50:6) | Positive | 840.71 | 8.76 |
| TG(50:6) | Positive | 840.71 | 8.67 |
| TG(50:6) | Positive | 840.71 | 9.49 |
| TG(51:1) | Positive | 864.80 | 10.21 |
| TG(51:2) | Positive | 862.78 | 9.81 |
| TG(51:3) | Positive | 860.77 | 9.47 |
| TG(51:4) | Positive | 858.75 | 9.18 |
| TG(51:4) | Positive | 858.75 | 10.07 |
| TG(51:4) | Positive | 858.76 | 10.56 |
| TG(52:0) | Positive | 880.83 | 11.04 |
| TG(52:1) | Positive | 878.81 | 10.46 |
| TG(52:2) | Positive | 876.79 | 9.99 |
| TG(52:3) | Positive | 874.78 | 9.62 |
| TG(52:4) | Positive | 872.77 | 9.30 |
| TG(52:5) | Positive | 870.75 | 9.05 |
| TG(52:5) | Positive | 870.75 | 9.16 |
| TG(52:6) | Positive | 868.73 | 8.80 |
| TG(52:6) | Positive | 868.74 | 9.02 |
| TG(52:6) | Positive | 868.74 | 9.81 |
| TG(53:1) | Positive | 892.83 | 10.63 |
| TG(53:2) | Positive | 890.81 | 10.18 |
| TG(53:3) | Positive | 888.79 | 9.77 |
| TG(53:4) | Positive | 886.78 | 9.44 |
| TG(53:4) | Positive | 886.79 | 9.91 |
| TG(53:4) | Positive | 886.78 | 10.47 |
| TG(53:4) | Positive | 886.79 | 11.04 |
| TG(53:5) | Positive | 884.77 | 9.16 |
| TG(53:5) | Positive | 884.77 | 9.55 |
| TG(53:5) | Positive | 884.77 | 9.99 |
| TG(54:0) | Positive | 908.85 | 11.60 |
| TG(54:1) | Positive | 906.84 | 10.92 |
| TG(54:2) | Positive | 904.83 | 10.36 |
| TG(54:3) | Positive | 902.81 | 9.91 |
| TG(54:4) | Positive | 900.79 | 9.55 |
| TG(54:5) | Positive | 898.78 | 9.26 |
| TG(54:5) | Positive | 898.78 | 9.46 |
| TG(54:6) | Positive | 896.77 | 9.01 |
| TG(54:6) | Positive | 896.77 | 9.16 |
| TG(54:7) | Positive | 894.75 | 8.78 |
| TG(54:8) | Positive | 892.74 | 8.74 |
| TG(54:8) | Positive | 892.74 | 8.57 |
| TG(55:1) | Positive | 920.86 | 11.17 |
| TG(55:2) | Positive | 918.84 | 10.58 |
| TG(55:2) | Positive | 918.85 | 10.41 |
| TG(55:3) | Positive | 916.83 | 9.76 |
| TG(55:3) | Positive | 916.83 | 10.11 |
| TG(55:4) | Positive | 914.81 | 9.74 |
| TG(55:4) | Positive | 914.81 | 9.52 |
| TG(56:0) | Positive | 936.89 | 12.25 |
| TG(56:1) | Positive | 934.87 | 11.45 |
| TG(56:2) | Positive | 932.86 | 10.80 |
| TG(56:3) | Positive | 930.84 | 9.86 |
| TG(56:3) | Positive | 930.84 | 10.27 |
| TG(56:4) | Positive | 928.83 | 9.85 |
| TG(56:5) | Positive | 926.81 | 9.77 |
| TG(56:6) | Positive | 924.80 | 9.41 |
| TG(56:6) | Positive | 924.80 | 9.26 |
| TG(56:7) | Positive | 922.78 | 9.12 |
| TG(56:7) | Positive | 922.78 | 9.24 |
| TG(56:7) | Positive | 922.78 | 9.00 |
| TG(56:8) | Positive | 920.76 | 8.99 |
| TG(56:9) | Positive | 918.75 | 8.74 |
| TG(58:1) | Positive | 962.90 | 12.08 |
| TG(58:10) | Positive | 944.76 | 8.74 |
| TG(58:10) | Positive | 944.76 | 8.87 |
| TG(58:11) | Positive | 942.75 | 8.54 |
| TG(58:2) | Positive | 960.89 | 11.31 |
| TG(58:3) | Positive | 958.87 | 10.71 |
| TG(58:4) | Positive | 956.86 | 10.23 |
| TG(58:5) | Positive | 954.84 | 9.81 |
| TG(58:5) | Positive | 954.84 | 9.93 |
| TG(58:5) | Positive | 954.85 | 10.06 |
| TG(58:6) | Positive | 952.83 | 9.69 |
| TG(58:6) | Positive | 952.83 | 9.52 |
| TG(58:6) | Positive | 952.83 | 9.83 |
| TG(58:7) | Positive | 950.81 | 9.36 |
| TG(58:7) | Positive | 950.81 | 9.49 |
| TG(58:7) | Positive | 950.81 | 9.23 |
| TG(58:8) | Positive | 948.79 | 9.22 |
| TG(58:8) | Positive | 948.79 | 9.09 |
| TG(58:8) | Positive | 948.79 | 8.98 |
| TG(58:9) | Positive | 946.78 | 8.97 |
| TG(58:9) | Positive | 946.78 | 8.85 |
| TG(60:10) | Positive | 972.79 | 8.95 |
| TG(60:10) | Positive | 972.79 | 9.13 |
| TG(60:11) | Positive | 970.78 | 8.83 |
| TG(60:2) | Positive | 988.92 | 11.92 |
| TG(60:3) | Positive | 986.91 | 11.16 |
| TG(60:9) | Positive | 974.814 | 9.19 |
| TG(60:9) | Positive | 974.81 | 8.95 |
