# Supplementary material for: Lipidomics of human adipose tissue reveals diversity between body areas
Source: PLoS One. 2020 Jun 16;15(6):e0228521. doi: 10.1371/journal.pone.0228521 (PMC7297320; doi:10.1371/journal.pone.0228521)
Supplement: S2 Table — (DOCX) [file pone.0228521.s003.docx]

**Table 2**. Identified lipids from Negative ionization mode.

| **Lipid Name** | **Ionization Mode** | ***Mass-to-charge ratio (m/z)*** | **Retention time (RT)** |
| --- | --- | --- | --- |
| Cer(d34:1) | Negative | 582.51 | 7.43 |
| Cer(d36:1) | Negative | 610.54 | 7.66 |
| Cer(d37:1) | Negative | 624.56 | 8.07 |
| Cer(d40:1) | Negative | 666.60 | 8.59 |
| Cer(d41:1) | Negative | 680.62 | 8.75 |
| Cer(d42:1) | Negative | 694.63 | 8.91 |
| Cer(d42:2) | Negative | 692.62 | 8.54 |
| FA(16:0) | Negative | 255.23 | 5.05 |
| FA(18:0) | Negative | 283.26 | 5.65 |
| FA(18:1) | Negative | 281.25 | 5.09 |
| LPC(16:0) | Negative | 540.33 | 4.09 |
| LPC(18:0) | Negative | 568.36 | 4.57 |
| LPC(18:1) | Negative | 566.34 | 4.11 |
| PC(32:0) | Negative | 778.56 | 7.41 |
| PC(34:1) | Negative | 804.57 | 7.39 |
| PC(34:2) | Negative | 802.56 | 6.99 |
| PC(36:1) | Negative | 832.60 | 7.81 |
| PC(36:4) | Negative | 826.56 | 6.88 |
| PC(P-34:0)/PC(O-34:1) | Negative | 790.60 | 7.69 |
| PC(P-34:2)/PC(O-34:3) | Negative | 786.56 | 7.21 |
| PE(34:1) | Negative | 716.52 | 7.54 |
| PE(36:0) | Negative | 746.57 | 7.82 |
| PE(36:0) | Negative | 746.57 | 8.29 |
| PE(38:4) | Negative | 766.54 | 7.44 |
| PE(O-34:3)/PE(P-34:2) | Negative | 698.51 | 7.34 |
| PE(O-36:3)/PE(P-36:2) | Negative | 726.54 | 7.76 |
| PE(O-36:5)/PE(P-36:4) | Negative | 722.51 | 7.19 |
| PE(O-38:6)/PE(P-38:5) | Negative | 748.53 | 7.19 |
| PE(O-40:5)/PE(P-40:4) | Negative | 778.57 | 7.97 |
| PG(36:3) | Negative | 817.52 | 7.56 |
| PS(36:2) | Negative | 786.52 | 7.95 |
| PS(38:5) | Negative | 854.51 | 7.95 |
| SHexCer(d34:2) | Negative | 776.49 | 7.95 |
