# Supplementary material for: Lipidomics of human adipose tissue reveals diversity between body areas
Source: PLoS One. 2020 Jun 16;15(6):e0228521. doi: 10.1371/journal.pone.0228521 (PMC7297320; doi:10.1371/journal.pone.0228521)
Supplement: S1 Fig — Statistics are grouped by the comparison pair (columns) and the lipid class (rows from DG to TG) into panels. Within each panel, each colored rectangle corresponds to one lipid species, and its location in the x-axis and y-axis, respectively, shows its level of unsaturation (number of double bonds) and its size (number of carbon atoms in the fatty acid chains). Blue, red and white rectangles, respectively, indicate lower, higher and same levels in thigh compared to abdomen (based on the regression coefficient from the linear regression model with the body area as an independent variable and the lipid level as the dependent variable. Statistical significance of the difference is annotated by the symbols “*,” “x” and “+,” respectively, corresponding to p < 0.01, 0.05 and 0.1. For instance the comparison between thigh and abdomen is the fourth column from the left. In that comparison, the triacylglycerol TG(60:11) is located in the x- and y-coordinates 11 and 60, respectively, (the top-rightmost corner) in the TG panel. The lipid TG(60:11) has a total of 60 carbon atoms and 11 double bonds (i.e., unsaturated bonds) in its fatty-acid chains. The lipid has a clearly higher level in thigh compared to abdomen (red color of the rectangle) with a statistical significance of p < 0.01 (annotation with the character “*”). (DOCX) [file pone.0228521.s004.docx]

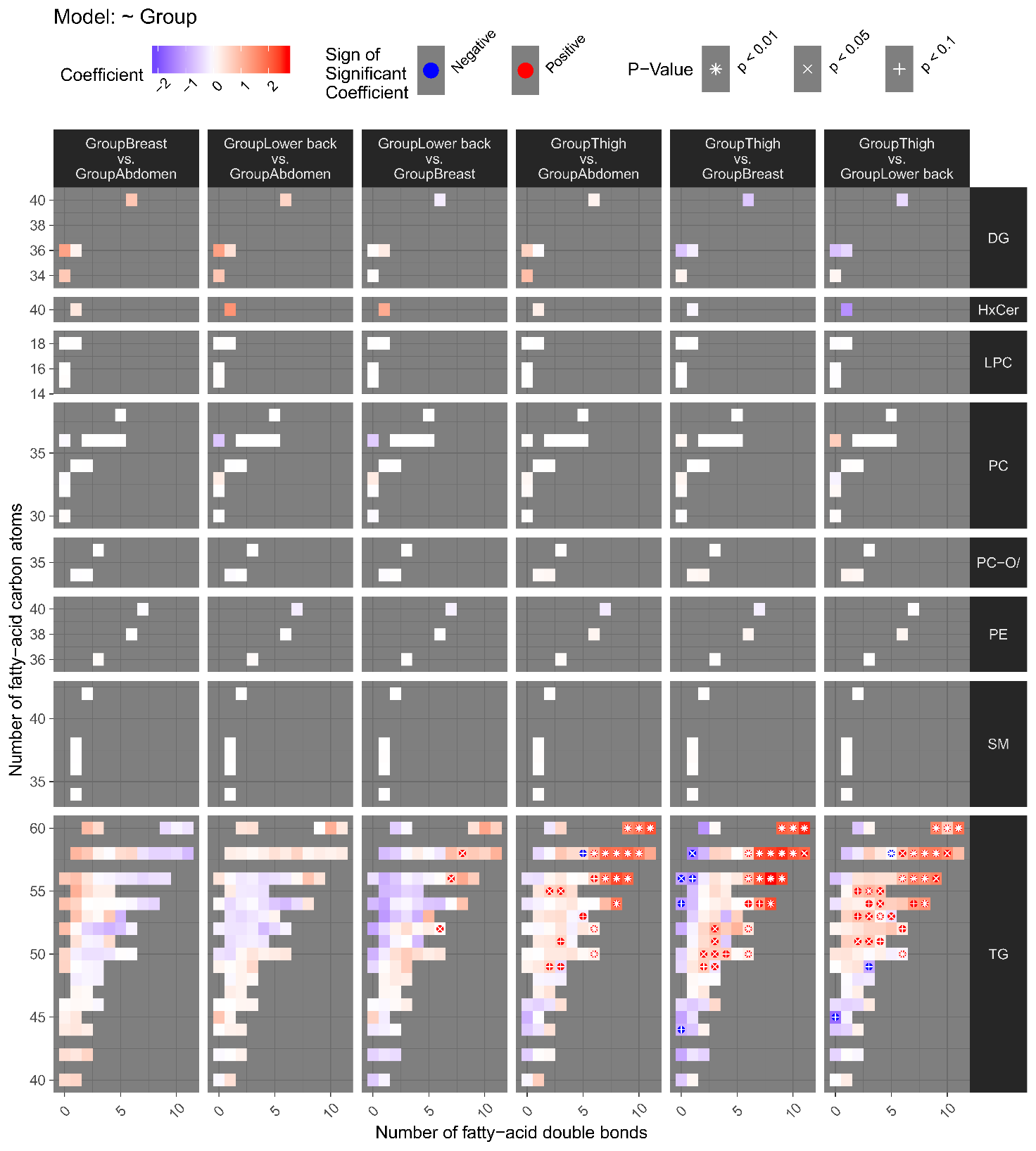


**Supplementary Figure 1**: Heatmap of all the comparisons of the lipidome between body areas. Statistics are grouped by the comparison pair (columns) and the lipid class (rows from DG to TG) into panels. Within each panel, each colored rectangle corresponds to one lipid species, and its location in the x-axis and y-axis, respectively, shows its level of unsaturation (number of double bonds) and its size (number of carbon atoms in the fatty acid chains). Blue, red and white rectangles, respectively, indicate lower, higher and same levels in thigh compared to abdomen (based on the regression coefficient from the linear regression model with the body area as an independent variable and the lipid level as the dependent variable. Statistical significance of the difference is annotated by the symbols “*,” “x” and “+,” respectively, corresponding to p < 0.01, 0.05 and 0.1. For instance the comparison between thigh and abdomen is the fourth column from the left. In that comparison, the triacylglycerol TG(60:11) is located in the x- and y-coordinates 11 and 60, respectively, (the top-rightmost corner) in the TG panel. The lipid TG(60:11) has a total of 60 carbon atoms and 11 double bonds (i.e., unsaturated bonds) in its fatty-acid chains. The lipid has a clearly higher level in thigh compared to abdomen (red color of the rectangle) with a statistical significance of p < 0.01 (annotation with the character “*”).
